# Oxytocin Signaling Acts as a Marker for Environmental Stressors in Zebrafish

**DOI:** 10.3390/ijms22147459

**Published:** 2021-07-12

**Authors:** Hsin-Ju Chuang, Chun-Yung Chang, Huai-Ping Ho, Ming-Yi Chou

**Affiliations:** Department of Life Science, National Taiwan University, Taipei 10617, Taiwan; d08b21004@ntu.edu.tw (H.-J.C.); chunyungc@ntu.edu.tw (C.-Y.C.); r09b21015@ntu.edu.tw (H.-P.H.)

**Keywords:** oxytocin system, oxytocin receptor, zebrafish, stress, environmental stressors, environmental markers

## Abstract

The oxytocin system plays a role in stress responses and behavior modulation. However, the effects of oxytocin signaling on stress adaptation remain unclear. Here, we demonstrated the roles of oxytocin signaling as a biomarker under stress conditions in the peripheral tissues (the gills) and central nervous system (the brain). All the environmental stressors downregulated the expression of oxytocin receptors in the gills, and the alteration of the expression of oxytocin receptors was also found in the brain after the acidic (AC) and high-ammonia (HA) treatments. The number of oxytocin neurons was increased after double-deionized (DI) treatment. By transgenic line, Tg(oxtl:EGFP), we also investigated the projections of oxytocin neurons and found oxytocin axon innervations in various nuclei that might regulate the anxiety levels and aggressiveness of adult zebrafish under different environmental stresses. The oxytocin system integrates physiological responses and behavioral outcomes to ensure environmental adaptation in adult zebrafish. Our study provides insight into oxytocin signaling as a stress indicator upon environmental stressors.

## 1. Introduction

Animals adapt to the external environment throughout their life. Environmental stimuli can be classified as biotic or abiotic. The stimuli that disturb the internal dynamic equilibrium are defined as stressors [1,2,3], and they include pH, nitrogenous waste, salinity, temperature, anoxia, pollutants, and predation [4,5,6,7,8,9,10,11,12]. Stress responses are conserved in vertebrates and are divided into three phases [13,14]. To cope with stress, in animals, the neuroendocrine system is first activated to secrete hormones that alter physiological and metabolic pathways. Subsequently, systemic changes occur, which may reverse the negative impact of stressors.

The hypothalamus plays a primary role in stress responses; it integrates sensory inputs, controls hormone secretion, and regulates various functions, including the readjustment of biological activity and energy allocation [3,15,16]. Many hormones have been reported to be involved in stress responses [17,18,19]. Among them, cortisol, which is regulated by the hypothalamic–pituitary–adrenal (HPA) axis, is the most studied stress indicator, and it promotes “fight and flight” abilities in fish [2,20]. In zebrafish, the knockdown of glucocorticoid receptor (GR) by morpholino (MO) downregulated the expression of acid-secreting transporters and acid secretion, which decreased the ability of the zebrafish to cope with acidic environments [17]. Intraperitoneal injection with cortisol upregulated the expression and activity of Na-K-ATPase in the gills of milkfish under hypotonic stress [21]. However, the cortisol concentration in the blood is not always increased during stress. The cortisol level in serum was decreased in yellow perch treated with multiple stressors, including chemical pollutants and capture stress [22]. In sea bream, only 80 µg/L gold nanoparticles coated with PVP (AuNP-PVP) upregulated cortisol levels in the plasma, and 4 and 1600 µg/L AuNP-PVP did not change plasma cortisol levels [23]. Furthermore, some moderate stressors are insufficiently strong to trigger cortisol release. Compared with 25 mg/L aluminum oxide (Al_2_O_3_), 1 and 5 mg/L Al_2_O_3_ did not change cortisol levels in the blood of tilapia [24]. To date, several hypothalamic hormones have been demonstrated to participate in stress responses [25].

Oxytocin is a polypeptide neurotransmitter/hormone that mediates uterus construction and milk ejection [26,27,28]. Recent studies have shown that hypothalamic oxytocin is involved in stress responses. In rodents, forced swimming stress and shaker stress induced the parallel release of oxytocin in the hypothalamus and peripheral circulation [29,30]. Oxytocin-knockout mice displayed anxiety-like behaviors and an increased corticosterone level in the blood after psychogenic stressor on a platform shaker or in novel environments [31]. In patients with generalized social anxiety disorder, higher plasma oxytocin levels led to higher social anxiety and dissatisfaction [32]. Furthermore, recent studies have clarified the regulatory role of oxytocin on glucose homeostasis. Glucose levels were elevated in the blood of oxytocin-knockout mice fed a high fat diet [33]. Oxytocin attenuated the peak value of plasma glucose in men after intranasal administration [34]. The oxytocin receptor is expressed in the amygdala [35], and this receptor mediates an increase in oxytocin release in stimulated fear-attenuating responses, which may help individuals better respond to urgent situations [36]. Oxytocin is present in the hypothalamus and is widely present in various tissues [37,38]; furthermore, it plays roles in stress responses [39]. The oxytocin system (oxytocin and oxytocin receptors) may serve as an indicator for evaluating whether the individual is under stress conditions.

Because of aquatic habitats, fish possess high tolerance and sensitivity to harsh environments compared with terrestrial organisms [14,40], making fish popular experimental animals for studies of environmental acclimation [12,41,42]. For example, the blood glucose level was increased in *Labeo capensis* (mudfish) and *Micropterus salmoides* (largemouth bass) after exposure to high-ammonia stress [43,44]. Cortisol concentrations in the blood were increased under hypo- or hyper-osmotic stress in milkfish and tilapia [21,45]. However, the roles of oxytocin in stress responses and the underlying mechanisms in fish are unclear; more data are required to elucidate this research gap.

To address this research gap, we used zebrafish (*Danio rerio*) as the animal model to investigate the roles of the oxytocin system under different environmental challenges. Previous studies indicated that the oxytocin controlled the social behaviors in the adult zebrafish [46,47]. However, the role of oxytocin in stress response is still unknown. Zebrafish possesses similar physiological responses compared to mammals/humans [48]. A small size, rapid lifecycle, and ability to be easily genetically manipulated make zebrafish convenient for many experimental designs. Various molecular physiological approaches can be applied to study the functions of zebrafish genes and cells, and many behavioral paradigms can be used to evaluate the emotional states and social interactions of adult zebrafish [49,50]. The main goal of the present study was to evaluate the potential of oxytocin system as a stress indicator and investigate the role of oxytocin in stress responses in the zebrafish. In present study, we exposed adult zebrafish to various environmental stresses and measured the expression of oxytocin receptors in the gills and brain. We investigated the numbers of oxytocin neurons in the brain under different environmental stresses. We also traced the projections of oxytocin neurons in the brain and performed behavioral tests to evaluate behavioral alteration under different environmental challenges. Hence, that the oxytocin system is a potential biomarker of the stress response in aquatic teleosts is an expected result of this study.

## 2. Results

### 2.1. Oxyr and Oxyrl Were Expressed in the Brains and Gills of Adult Zebrafish

**Figure 1:**
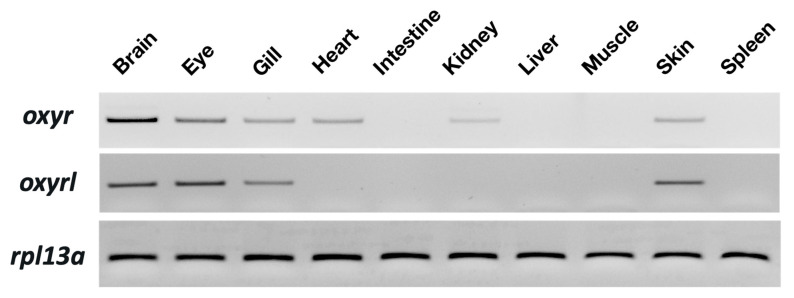
Distribution of *oxyr* and *oxyrl* in different tissues of adult zebrafish. The mRNA expression of target genes was analyzed through semi-quantitative PCR; *rpl13a* was used as an internal control to confirm the cDNA quality of different tissues.

Tissue distribution of *oxyr* and *oxyrl* in the zebrafish was examined through semi-quantitative PCR with 40 amplification cycles. *Rpl13a* was used as an internal control to confirm the equal abundance of RNA in various tissues. *Oxyr* showed the highest expression in the brain and moderate expression in the eyes, gills, hearts, intestines, and skin of the adult zebrafish (Figure 1). In the kidneys, a weak signal of *oxry* was detected. For *oxyrl*, the expression in the eyes and brains was the highest, and moderate signals were found in the gills and skin of the adult zebrafish (Figure 1). 

### 2.2. Environmental Stressors Downregulated Oxyr mRNA Expression in the Gills

**Figure 2:**
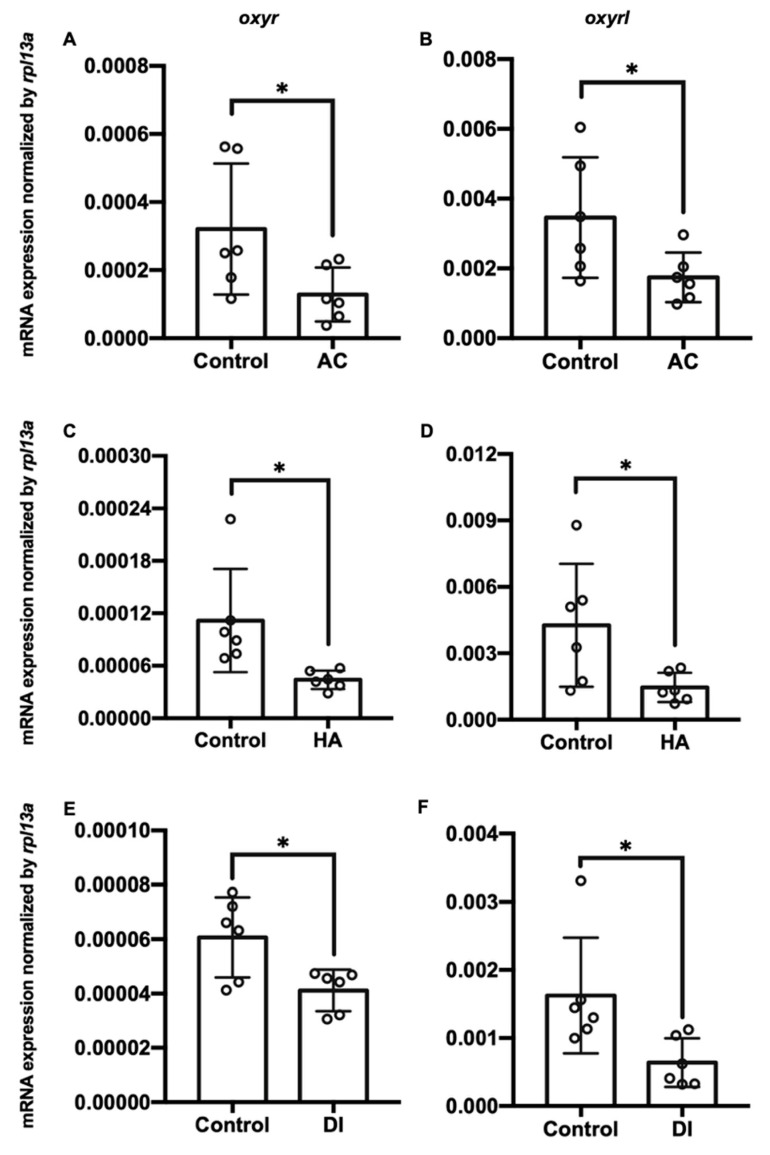
Charts showing mRNA expression of *oxyr* and *oxyrl* in the gills of adult zebrafish acclimated to (**A**,**B**) acidic (AC), (**C**,**D**) high-ammonia (HA), or (**E**,**F**) double-deionized (DI) water for 7 days. *Rpl13a* was used as an internal control to normalize relative expression. Each circle represents the data of one single fish. The asterisks (*) indicate significant differences between the control and treatment groups. Values are means ± SD (*n* = 6, *p* < 0.05 (Student’s *t*-test)).

**Figure 3:**
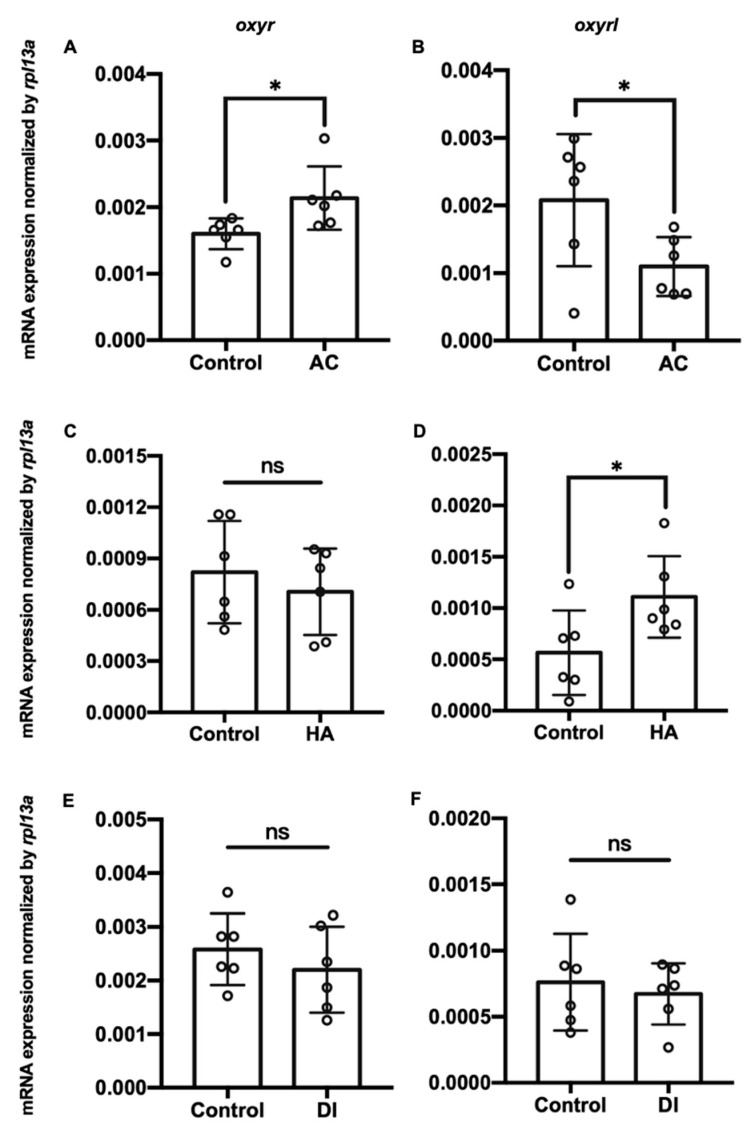
Charts showing mRNA expression of *oxyr* and *oxyrl* in the brains of adult zebrafish acclimated to (**A**,**B**) AC; (**C**,**D**) HA, or (**E**,**F**) DI water for 7 days. *Rpl13a* was used as an internal control to normalize relative expression. Each circle represents the data of one single fish. The asterisks (*) indicate significant differences between the control and treatment groups; ns indicates that no significant difference was found between the control and treatment groups. Values are means ± SD (*n* = 6, *p* < 0.05, (Student’s *t*-test)).

Three environmental challenges, namely acidic (AC), high-ammonia (HA), and double-deionized (DI) water, were applied to investigate the effects of environmental fluctuations on *oxyr* and *oxyrl* expression in the adult zebrafish. After the exposure of the zebrafish to these challenges, their gills and brains were dissected, and the total RNA of these two tissues was extracted for RT-qPCR analysis. AC, HA, and DI treatments significantly downregulated the mRNA expression of *oxyr* and *oxyrl* in the gills (Figure 2). After exposure to AC water for 7 days, the mRNA expression of *oxyr* and *oxyrl* in the gills declined by approximately 54% and 66% compared with that in the control fish gills, respectively (Figure 2A,B); HA treatment reduced the expression of *oxyr* and *oxyrl* in the gills by 61% and 69% for 7 days, respectively (Figure 2C,D). DI treatment caused 32% and 63% reductions in *oxyr* and *oxyrl* mRNA expression in the gills, respectively, compared with that in the control fish gills (Figure 2E,F). In the brain, *oxyr* expression increased by 38% after AC water exposure for 7 days, whereas *oxyrl* expression decreased by 58% after AC treatment (Figure 3A,B). After exposure to HA water, *oxyr* expression in the brain was similar to that in the control fish brains, and HA treatment significantly upregulated *oxyrl* expression by 83% (Figure 3C,D). DI treatment did not affect the expression of *oxyr* and *oxyrl* in the brain (Figure 3E,F).

### 2.3. DI Water Elevated the Number of Oxytocin Neurons in the Brain

**Figure 4:**
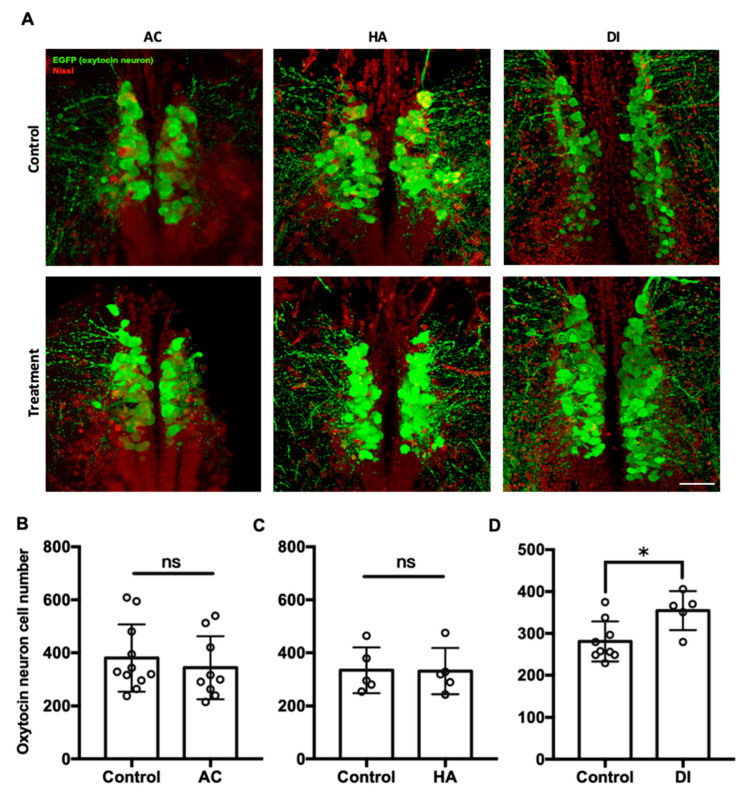
Confocal laser scanning micrographs of the sections of the brain of transgenic zebrafish (oxtl:EGFP) acclimated to AC, HA, or DI water stained with anti-GFP (green; oxytocin) and Nissl (red) (**A**). Scale bar = 50 µm. The total numbers of oxytocin neurons in the brain of transgenic zebrafish treated with AC (**B**), HA (**C**), and DI (**D**) water were counted through the section sets from whole brain. Each circle represents the data from one single fish. In AC treatment, *n* = 11 for control group; *n* = 9 for the treatment group. In HA treatment, *n* = 5 for both control and treatment group. In DI treatment, *n* = 9 for control group; *n* = 5 for the treatment group. The asterisks (*) indicate significant differences between the control and treatment groups; ns indicates that no significant difference was found between the control and treatment groups. Values are mean ± SD (*p* < 0.05 (Student’s *t*-test)).

To investigate the effects of environmental stressors on the oxytocin system in the brain, the transgenic zebrafish Tg(oxtl:EGFP) were used, in which oxytocin neurons and their projections are labeled. In Figure 4, each circle represented the number of oxytocin neuron number in the brain of a single individual. After exposure to AC water for 7 days, the number of oxytocin neurons in the preoptic area of the brain was similar between the AC group (343.7 ± 118.6) and the control group (380.1 ± 127.2) (Figure 4B). Similarly, HA treatment did not affect the number of oxytocin neurons in the brain (HA group, 330.7 ± 87.3; control group, 334.2 ± 86.4) (Figure 4C). After DI treatment for 7 days, the number of oxytocin neurons in the DI group (476.6 ± 63.7) was significantly higher than that in the control group (378.3 ± 35.8) (Figure 4D).

### 2.4. Mapping Central Projections of Oxytocin Neurons in Adult Zebrafish

**Figure 5:**
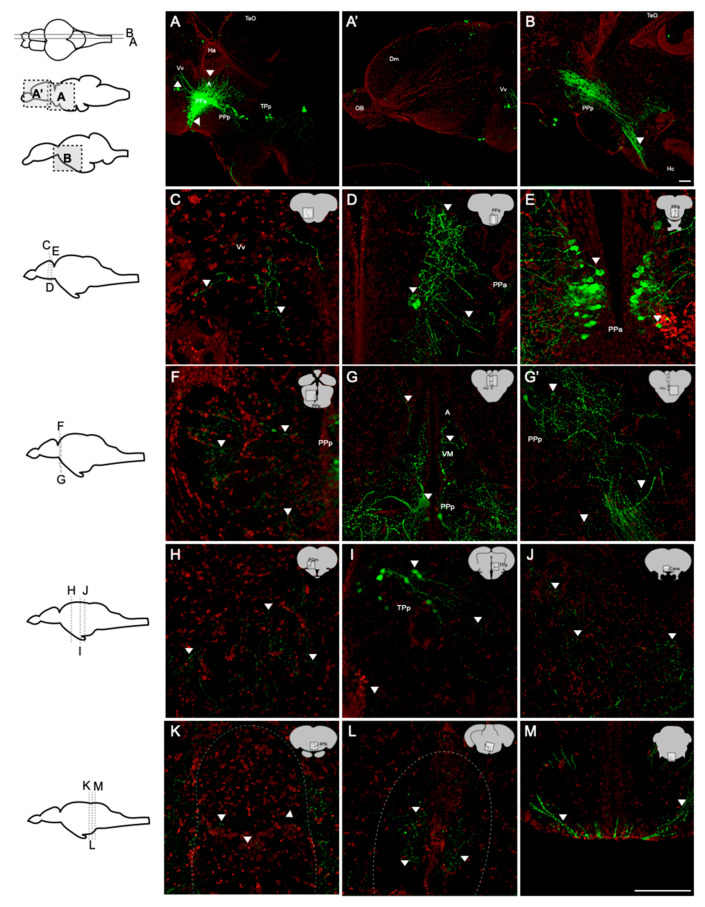
Distribution and projection of oxytocin neurons in the adult zebrafish brain (*n* = 5). Whole-brain sections were stained with anti-GFP (green; oxytocin) and Nissl (red). (**A**,**B**) Sagittal sections showed that the fibers of oxytocin neurons were widely present in the brain. Somas were found in (**D**,**E**) PPa, (**F**,**G**) PPp, and (**I**) TPp. From the rostral end to the caudal end, the regions containing the fibers were found in (**C**) Vv, (**D**,**E**) PPa, (**F**) PPp, (**G**) A, VM, (**H**) PGm, (**I**) TPp, (**J**) Cans, (**K**) IPN, (**L**) raphe, and (**M**) TTBc. Scale bar = 100 µm.

To investigate the projections of oxytocin neurons in the brain of the adult zebrafish, the whole-brain sections of a transgenic zebrafish line, Tg(oxtl:EGFP), was used to visualize the cell bodies of oxytocin neurons and their projections. Immunohistochemistry of GFP was performed to enhance green fluorescent signals. In the sagittal brain sections, the cell bodies of oxytocin neurons were mainly located in the parvocellular preoptic nucleus including the anterior part (PPa) and the posterior part (PPp) (Figure 5A,B). Few neurons were also found in the periventricular nucleus of the posterior tuberculum (TPp), and these neurons were smaller than those in the parvocellular preoptic nucleus (Figure 5A). Oxytocin neurons in the PPa and PPp sent a high density of axons toward the caudal zone of the periventricular hypothalamus (Hc) to the pituitary gland (Figure 5B). Oxytocin neurons also projected to the ventral nuclei of the ventral telencephalic area (Vv) (Figure 5A) but not to the olfactory bulb (Figure 5A). To observe the innervation of oxytocin neurons more clearly, we performed coronal sectioning using a vibratome. In the telencephalon, few fibers of oxytocin neurons were found in the Vv (Figure 5C), and many fibers were observed in the PPa (Figure 5D) as well as many oxytocin neuron cell bodies (Figure 5E). In the midbrain, few cell bodies were found in the PPp, and these neurons sent axons dorsally to the anterior and ventromedial thalamic nuclei (A and VM) (Figure 5F,G) and ventral-caudally to the pituitary (Figure 5G). Few cell bodies were also found in the TPp (Figure 5I). In the preglomerular nuclei (PGm), TPp, commissura ansulata (Cans), interpeduncular nucleus (IPN), and raphe, the signals of oxytocin axons were found (Figure 5H–L). In the hindbrain, oxytocinergic axons extended to the crossed tecto-bulbar tract (TTBc; Figure 5M). No cell body was found in the hindbrain.

### 2.5. Effects of Environmental Stressors on Swimming and Aggressive Behaviors

**Figure 6:**
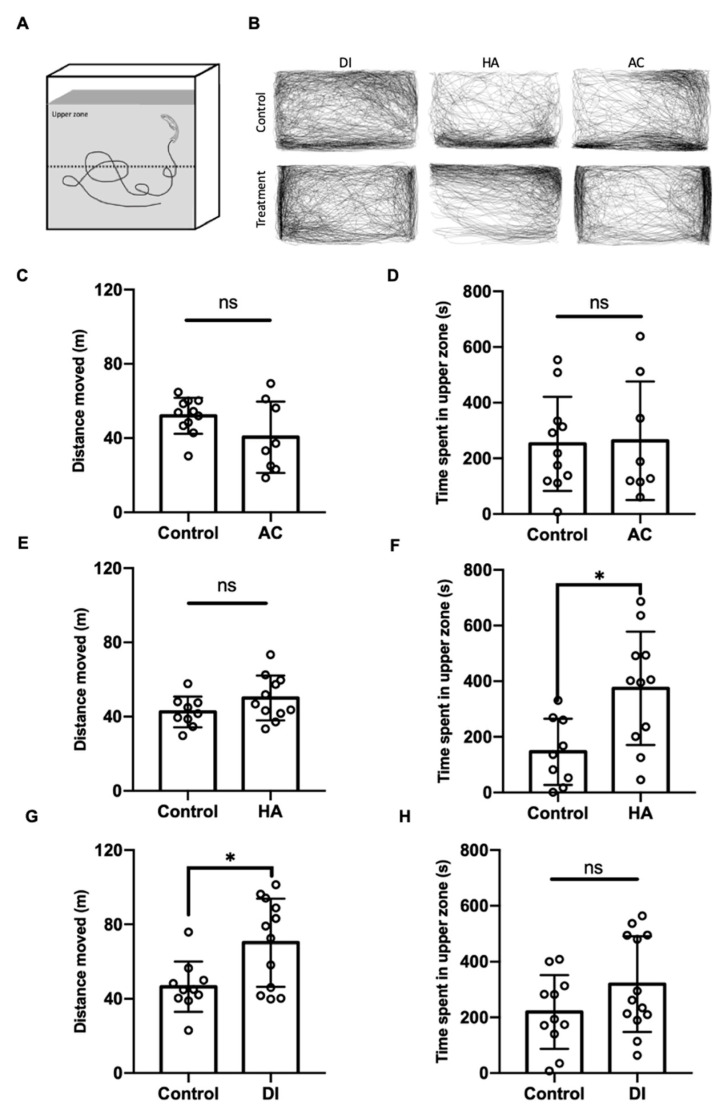
Effects of AC, HA, and DI treatments in a novel tank diving test. (**A**) Schematic of the novel tank diving test. (**B**) Trajectories of fish moving after exposure to AC, HA, or DI water. (**C**,**E**,**G**) Total moving distance of fish was calculated after exposure to different stressors. (**D**,**F**,**H**) The period fish stayed in upper zone was analyzed after exposure to different stressors. In AC treatment, *n* = 11 for the treatment group, *n* = 8 for the treatment group. In HA treatment, *n* = 9 for control group; *n* = 11 for the treatment group. In DI treatment, *n* = 11 for control group; *n* = 13 for the treatment group. The asterisks (*) indicate significant differences between the control and treatment groups.; ns indicates that no significant difference was found between the control and treatment groups. Values are means ± SD (*p* < 0.05 (Student’s *t*-test)).

To evaluate the anxiety level of the adult zebrafish under various environmental stresses, the novel tank diving test was performed. After exposure to DI water for 24 h, the zebrafish’s total moving distance was significantly increased, but the time spent in the upper part of the tank was similar to that of the control fish (Figure 6G,H); by contrast, after exposure to HA water for 24 h, relative to the control fish, the fish spent more time in the upper part of tank but showed a similar moving distance (Figure 6E,F). AC treatment did not affect the anxiety level and locomotion activity of the fish (Figure 6C,D).

**Figure 7:**
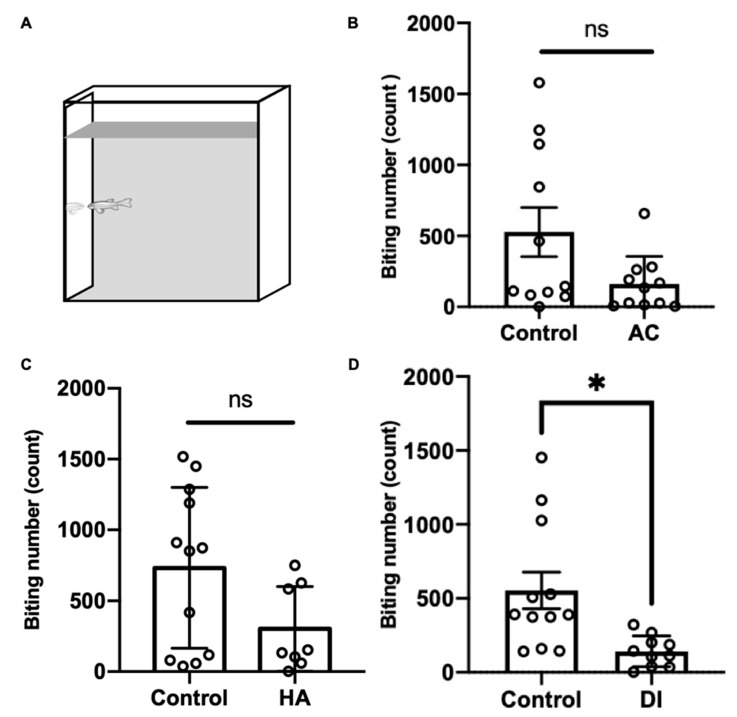
Effects of AC, HA, and DI treatments in the mirror biting test. (**A**) Schematic of the mirror biting test. Adult zebrafish were exposed (**B**) AC, (**C**) HA, or (**D**) DI, and the mirror-biting frequency was determined. Each circle represents the data of one single fish. In AC treatment, *n* = 11 for control group; *n* = 10 for the treatment group. In HA treatment, *n* = 12 for control group; *n* = 8 for the treatment group. In DI treatment, *n* = 11 for control group; *n* = 10 for the treatment group. All the treatments reduced mirror-biting frequency relative to control fish. The asterisks (*) indicate significant differences between the control and treatment groups.; ns indicates that no significant difference was found between the control and treatment groups. Values are means ± SD in (**A**) and (**C**) (*p* < 0.05 (Student’s *t*-test)). Values are means ± SEM in (**B**) (*p* < 0.05 (Mann–Whitney)).

To evaluate the aggressiveness of the adult zebrafish under various environmental stresses, the mirror biting test was performed. After AC, HA, and DI water treatments for 24 h, the fish showed a lower biting frequency than did the control fish; however, the finding did not reach statistical significance in the AC and HA groups (Figure 7).

## 3. Discussion

The present study demonstrated that the oxytocin system participated in the acclimation of zebrafish to environmental fluctuations. The expression of oxytocin receptors in the peripheral tissue (the gills) and the central nervous system (the brain) was altered by environmental stresses; this finding indicates that the oxytocin system serves as a stress indicator. We also provided anatomical and behavioral evidences induced by stressors through the oxytocin system. This study evaluated the role of oxytocin signaling as a potential stress indicator, and our study results provided insights into the effects of oxytocin signaling on stress responses.

Oxytocin is a conserved neuropeptide with multiple functions, including adaptation to stress [39,51]. Oxytocin in the central nervous system reduced corticosterone in rats exposed to noise stress [52]. A higher plasma oxytocin level was discovered in older women under social stress [51]. Moreover, recent studies have indicated that the expression and functions of oxytocin receptors are important for adaptation to stress among animals. Forced swimming stress increased the expression of oxytocin receptors in the brains of rats [53], whereas isolation stress downregulated the mRNA expression of oxytocin receptors in mice [54]. In humans, the polymorphism of oxytocin receptor genes is related to empathy and stress reactivity [55]. The expression of oxytocin receptors was upregulated by hypomethylation in patients with social anxiety disorder [56]. These findings indicate that oxytocin signaling is involved in stress responses. In the present study, the expression of two oxytocin receptor genes (*oxyr* and *oxyrl*) in the gills was downregulated by AC, HA, and DI treatments (Figure 2). Studies have demonstrated that physiological biomarkers (stress markers) are changed (either stimulated or suppressed) by stressors [3,13]. In tilapia, pollution stress upregulated the levels of cortisol, blood urea nitrogen, and thyroxine (T4) and downregulated the levels of blood glucose, triglyceride, and triiodothyronine (T3) [24]. Multiple stresses (netting, chasing, air exposure, and confinement stresses) increased plasma glucose and lactate levels in common carp [57]. Nickel (Ni) exposure decreased the levels of total protein, lipids, glucose, and glycogen in the gills of carp [58]. Chasing or netting stress upregulated the levels of cortisol, glucose, and lactate in the gills of zebrafish and rainbow trout [59]. In addition to physiological parameters, stress-related molecules are applied to evaluate whether individuals are under stress conditions. Hyperthermal stress caused an increase in heat shock protein 70 (HSP70) and a decrease in GR in rainbow trout [60]. Under hypertonic stress, the expression of HSP70 and HSP90 was upregulated in the gills of milkfish [12]. Under cold stress, the expression of GRs and mineralocorticoid receptors was downregulated in carp [61]. All the aforementioned observations indicate that the expression of molecular biomarkers reflect the stress conditions of fish. According to the aforementioned findings, the alteration of *oxyr* and *oxyrl* expression in the gills provides evidence that oxytocin signaling serves as a biomarker for stress conditions.

In the present study, the highest expression of oxytocin receptors was found in the brain (Figure 1). AC water induced *oxyr* expression in the brain (Figure 3A), and *oxyrl* expression was downregulated by AC water but upregulated by HA treatment (Figure 3B,D). The different expression patterns of *oxyr* and *oxyrl* in the brain under AC treatment revealed the different mechanisms of these two receptors. After DI treatment, the expression of *oxyr* and *oxyrl* did not change (Figure 3E,F). To investigate the effects of various environmental stresses on the oxytocin system, we measured the numbers of oxytocin neurons after various stress challenges. A previous study found that the cell bodies of oxytocin neurons were located in the PPa in rainbow trout [62]. In the present study, we observed oxytocin neuron somas in the parvocellular preoptic nucleus (PPa and PPp) and periventricular nucleus of TPp (Figure 4 and Figure 5). The oxytocin neuron numbers increased after DI treatment but remained unchanged after AC and HA treatments (Figure 4). In mice, repeated defeat experiences and vicarious social stress increased the number of oxytocin neurons in the paraventricular nucleus of the hypothalamus (PVN) and bed nucleus of the stria terminalis, respectively [63,64]. Under chronic homotypic stress, the number of oxytocin neurons in the PVN also increased [65,66]. Given that the expression of oxytocin receptors in the brain changed according to various stressors and that the number of oxytocin neurons increased under hypotonic stress, the oxytocin system may be involved in acclimation to environmental fluctuations.

In addition to hormones, oxytocin serves as a neurotransmitter [67]. Oxytocin receptors are widely present in many brain regions of mammals, including the hypothalamus, prefrontal cortex (PFC), hippocampus, and amygdala [39,68]. The innervations of oxytocin neurons were observed in the cortex, pallidum, thalamic area, striatum, hypothalamic area, midbrain, hindbrain, and medulla of mice [69]. Through single-cell labeling and neurite tracing, Wircer and colleagues (2017) reported that zebrafish oxytocin neurons projected to the hindbrain and spinal cord 5 days post-fertilization (dpf) embryos [70]. Besides the projection of oxytocin neurons to hindbrain and spinal cord, Wee and her colleagues further demonstrated that this innervation of oxytocin neurons drove the nocifensive behavior of zebrafish via the premotor targets in the brainstem of 6–8 dpf embryos [71]. However, the projections of oxytocin neurons in the adult brain remained unclear. In the present study, for the first time, we observed oxytocin axons in the telencephalic area (Vv), anterior and ventromedial thalamic nuclei (A and VM), preglomerular nuclei (PGm), TPp, Cans, IPN, and raphe in the adult zebrafish brain (Figure 5). The projections and innervations of oxytocin neurons might regulate fish behaviors.

Oxytocin exerts anxiolytic and stress-alleviating effects in animals under stress [72]. Serotonergic neurons in the median raphe express oxytocin receptors and are activated by oxytocin, and such activation reduces anxiety-related behavior in mice [73]. Medial PFC (mPFC) interneurons expressing oxytocin receptors also exert anxiolytic effects by producing corticotropin-releasing hormone-binding protein that blocks the effects of corticotropin-releasing hormone [74]. The intranasal administration of oxytocin attenuated the activity of the amygdala in patients shown a fearful face [75]. The activation of presynaptic oxytocin receptors enhanced depolarization-evoked glutamate release in the hippocampus and reduced anxiety- and depressive-like behaviors [76]. Moreover, oxytocin neurons have been reported to project into the raphe, cortex areas, amygdala, and hippocampus [69], suggesting that the reduction in anxiety and fear responses is due to the oxytocin neuron innervation in these brain regions. Knobloch and colleagues (2012) demonstrated that hypothalamic oxytocin neurons projected to the central amygdala and that optogenetic stimulation of these neurons inhibited the output neurons of the central amygdala and decreased the freezing response after fear conditioning in rats [36]. In the present study, the axons of oxytocin neurons innervated the telencephalon and thalamus in adult zebrafish (Figure 5C,G, respectively). The telencephalon of fish is believed to be homologous to the mammalian limbic system, which controls anxiety, emotion, and motivation [77]. In zebrafish, the activation of cannabinoid receptor type-1 (CB1 receptor) in the telencephalon decreased anxiety from acute restraint stress [78]. Pharmacological activation of the GABAergic system in the telencephalon also reduced the anxiety levels of Japanese medaka [79]. The thalamus consists of several nuclei and is involved in various functions, including the regulation of anxiety and fear in humans [80]. Pharmacological suppression of c-fos expression in the reuniens (RE) and rhomboid (RH) nuclei in the thalamus increased avoidance/defensive behaviors in the elevated plus-maze in rats [81]. The abnormal asymmetry of the thalamic volume was demonstrated to be related to social anxiety disorder in children and adolescents under chronic family stress [82]. In the present study, HA treatment decreased the anxiety level in fish (Figure 6F). This result together with the finding that oxytocin neurons innervated the telencephalon and thalamus in the adult zebrafish brain provides evidence of the anxiolytic effects of oxytocin neuron innervation in specific brain regions under HA stress.

Through aggressive behavior, animals consolidate resources and status in society [83]. Oxytocin was reported to mediate aggression and affiliative behaviors in several species. Oxytocin-knockout mice showed reduced attacking time to their opponents but exhibited normal sensorimotor performance [84]. GABAergic neurons expressing oxytocin receptors in the lateral and capsular division of the central amygdala (CeL/C) inhibited the output of the medial part of the central amygdala (CeM) as well as decreased aggression toward pups and then enhanced the attacks toward intruders [85,86]. The intranasal administration of oxytocin in humans promoted an aggressive choice of fictitious partners in monetary games [87] and increased the inclination toward intimate partner violence in participants prone to physical aggression [88]. In zebrafish, the habenula–IPN circuit regulates aggression and social conflict [89,90]. The medial subregion of the dorsal habenula (dHbM)–intermediate/ventral IPN (i/vIPN) pathway and the lateral subregion of the dorsal habenula (dHbL)–dorsal/intermediate IPN (d/iIPN) pathway facilitated loser and winner behaviors through projections into the medial raphe and dorsal tegmental area, respectively, in male zebrafish [89]. Hunger potentiated the dHbL–d/iIPN pathway and increased the probability of winning a fight during dyadic fighting [90]. In addition, the raphe was reported to control the escalation of aggression. Pharmacological activation of presynaptic GABA_B_ receptors inhibited 5-HT neurons in the dorsal raphe nucleus (DRN) and enhanced aggressive behaviors in mice [91]. Microinjection of L-glutamate into the DRN dose-dependently upregulated the number of attacking bites toward intruders, and the glutamate level in the DRN was increased during aggressive encounters [92]. Optogenetic stimulation of the median raphe decreased aggression in a phasic-like manner, whereas the stimulation of the dorsal raphe marginally but continuously diminished aggression and promoted social interactions in mice [93]. In the present study, the axons of oxytocin neurons were found to reach the IPN and raphe of the adult zebrafish brain (Figure 5K,L, respectively). We also found that environmental fluctuations downregulated the aggressiveness of the fish (Figure 7). Altogether, these findings suggest that the innervation of oxytocin neurons modified neural transmission and/or plasticity in the IPN and raphe and subsequently reduced aggressiveness in the adult zebrafish under AC, HA, and DI water treatments.

## 4. Materials and Methods

### 4.1. Experimental Animals

Mature zebrafish (*D. rerio*, AB strain and transgenic line) were reared in tanks filled with circulating filtered tap water at 28 °C ± 1 °C that was partially refreshed periodically. They were reared under a photoperiod cycle of 14-h light:10-h darkness (9:00–23:00) [94], and the pH value of water was monitored and maintained at 7.2 ± 2. The transgenic line (oxtl:EGFP) was obtained from Gil Levkowitz’s laboratory, and the location of oxytocin neurons was determined in the fish [95]. All experiments were conducted according to the principles and procedures approved by the Institutional Animal Care and Use Committee of National Taiwan University (IACUC approval no. NTU107-EL-00205 to M.Y. Chou).

### 4.2. Environmental Challenges to Adult Zebrafish

The zebrafish were gently transferred from their home tanks to different extreme environments at 28 °C ± 1 °C for 7 days. During the experiments, the fish were housed at a density of 1 fish/L in independent 10 L tanks with aeration and starved to avoid the effects of feeding and there were the independent control groups for different treatments. At the same time, both male and female zebrafish were existed in the same tank (sex ratio about 50%). In the tanks, 8 L water was changed daily to maintain environmental stability. In the present study, acidic (AC) water, high-ammonia (HA) water, and double-deionized (DI) water were used as extreme environments. DI water was collected from a water-purification system (A4-A8SL, Prema, Taipei, Taiwan). HA water (water containing 5 mM NH_4_^+^) was prepared by adding 5 mL of stock solution (1 M NH_4_Cl) and 300 mM 3-(N-morpholino) propanesulfonic acid buffer (MOPS; K35208029, Merck, Darmstadt, Germany; the pH value stabilizer) to 1 L fresh water (FW, filtered tap water). AC water was prepared through the addition of concentrated H_2_SO_4_ to FW (pH 7.2); the pH of this acidic water was adjusted to 4 ± 0.1. Measurement of pH was conducted by using a portable pH meter (pH 3310, WTW GmbH, Weilheim, Germany). For DI and AC treatments, FW was used as the control. For HA, FW with 300 mM MOPS was used as the control.

### 4.3. Total RNA Isolation and Complementary DNA Preparation

Tissues were frozen in liquid nitrogen and then homogenized in an appropriate amount of TRIzol reagent (Ambion, Woodward, TX, USA), using TissueLyser II (Qiagen, Valencia, CA, USA), and total RNA was purified by following the manufacturer’s protocol. To remove genomic DNA, total RNA was treated with DNase I (Promega, Madison, WI, USA), and the quality and quantity of total RNA were determined through agarose gel electrophoresis and NanoDrop ND-1000 (Thermo Scientific, Wilmington, DE, USA), respectively. For complementary DNA (cDNA) preparation, SuperScript IV reverse transcriptase (Invitrogen, Carlsbad, CA, USA) was used for the conversion of 5 μg of total RNA by following the manufacturer’s protocol, and the cDNA was stored at −20 °C for further experiments.

### 4.4. mRNA Expression of Oxytocin Receptors

Total RNA of the following tissues was isolated from the adult zebrafish: brain, eyes, gills, heart, intestine, kidney, liver, muscle, skin, and spleen. Tissue distributions of the following genes were determined (with the encoded proteins given in parentheses): *oxyr* (oxytocin receptor), *oxyrl* (oxytocin receptor–like), and *rpl13a* (ribosomal protein L13a), which served as the internal positive control. The sequences and Ensemble ID of primers are listed in Table 1. The genes were detected through semi-quantitative polymerase chain reaction (PCR) with Q5 high-fidelity DNA polymerase (M0491L, New England BioLabs, Ipswich, MA, USA) by following the manufacturer’s protocol. Different reaction cycles and amount of templates were tested to make sure the sampling condition was in a linear/quantifiable range (data not shown), and the amplified conditions were finally decided as 40 cycles. All the amplicons were sequenced to confirm the predicted products.

### 4.5. Real-Time Quantitative Polymerase Chain Reaction

Real-time quantitative PCR (RT-qPCR) was performed by using a LightCycler 480 real-time PCR system (Roche, Penzberg, Germany) in a final volume of 10 μL. The reaction mixture consisted of 5 μL 2× SYBR Green I Master mix (Roche) and 500 nM primer pairs. The reaction conditions were as follows: preincubation at 95 °C for 5 min and 45 cycles of amplification at 95 °C for 10 s, 60 °C for 10 s, and 72 °C for 10 s. The primer sets used in RT-qPCR are provided in Table 1. Moreover, *rpl13a* and *β-actin* were used as internal controls to obtain the confident quantification [96]. In the present study, normalization through the use of two reference genes showed similar patterns; therefore, only data normalized to *rpl13a* are shown. The products from each primer pair were subjected to melting-curve analysis and Sanger sequencing to verify their specificity. The efficiency of amplification was confirmed through serial dilution of cDNA for each primer set [97]. A non-template control (NTC) was conducted with sterile water to determine background DNA contamination. The relative expression of target genes normalized to internal control [98] was determined by using the following equation: 2^−ΔΔC(t)^.

### 4.6. Brain Fixation and Sectioning

Transgenic zebrafish (oxtl:EGFP) were anesthetized on ice, and the heads of fish were removed immediately and fixed with 4% paraformaldehyde in phosphate-buffered saline (PBS, 137 mM NaCl, 2.7 mM KCl, 1.8 mM KH_2_PO_4_, and 10 mM Na_2_HPO_4_; pH 7.4) at 4 °C, overnight. After fixation, the skull was removed carefully, and the whole brain was then mounted in 2% agarose prepared with PBS. Subsequently, 45 and 60 µm sections were obtained by using a Vibratory Microtome (Leica VT1200 S, Leica Microsystems, Wetzlar, Germany), and the sections were maintained in PBS and stored at 4 °C for analysis.

### 4.7. Immunohistochemistry

The sections were rinsed with PBS-Tr (PBS with 0.1% Triton X-100; T8787, Sigma-Aldrich, UK) three times and then incubated in 1% blocking reagent (11096176001, Roche) diluted with PBS for at least 2 h, at room temperature.

For mapping the projections of oxytocin neurons, 60 µm–thick sections were incubated with rabbit anti-GFP antibody (1: 2000; ab13970) at 4 °C, overnight, followed by incubation with CF 568 goat anti-rabbit secondary antibody (20102; Biotium, Fremont, CA, USA), at room temperature, for 2 h. After the section was rinsed with PBS-Tr, it was reacted with NeuroTrace 640/660 (N21483, Invitrogen) and then incubated with 1 µg/mL DAPI for 20 min for nucleus targeting.

For cell counting, 45 µm–thick sections were incubated with rabbit anti-GFP antibody (1: 2000; ab13970) at 4 °C, overnight, followed by incubation with CF 488 goat anti-rabbit secondary antibody (20012; Biotium), at room temperature, for 2 h. After the section was rinsed with PBS-Tr, it was reacted with NeuroTrace 530/615 (N21482, Invitrogen); then cell counting was conducted.

### 4.8. Cell Counting for Oxytocin Neurons

Whole-brain sections were applied to quantify the number of oxytocin neurons in a single individual. About 10 sections were collected from one brain. The free software Fiji was used to analyze the images of each section that was taken by a Zeiss Axio Imager Z2 Upright Microscope equipped with a Prime 95B sCMOS camera (TELEDYNE Photometrics, Tucson, AZ, USA). The final cell-counting data were corrected by the thickness of sections and the diameter of the nuclei through Abercrombie’s equation [99].

### 4.9. Novel Tank Diving Test

The novel tank diving test was performed to measure the locomotion activity and anxiety level of the fish [100], and the procedure in our previous study was followed to ensure a consistent behavioral paradigm [94]. After exposure to DI, HA, and AC water for 24 h, the adult zebrafish were transported individually from their home tank to a 3.5 L tank (26 cm × 8 cm× 17 cm; water volume: 2.5 L), through careful handling, to reduce stress. Fish behaviors were recorded by a digital camera (SONY HDR-SR11, Tokyo, Japan), which was mounted at the lateral site of the test tank, and the videos were analyzed by using EthoVision XT 14 (Noldus Information Technology, Wageningen, The Netherlands). During the behavioral record, the tank was in an independent room without any interference to avoid the effects from surrounding area. The center of gravity of the fish was defined as the fish position. The total swimming distance and the time spent at the top of the tank were analyzed for 15 min after transfer of the fish to evaluate the locomotion activity and anxiety level. The test was repeated thrice.

### 4.10. Mirror-Biting Test

The mirror-biting test was used to measure aggressiveness [100] according to a previously described method [94]. A 3.5 L tank (26 cm × 8 cm × 18 cm; water volume: 2.5 L) with a mirror attached to one of its sidewalls was used. Before the fish were placed in the test tank, an opaque partition was placed to prevent the fish from seeing the mirror. After exposure to AC, HA, and DI water for 24 h, the zebrafish were carefully transferred individually from their home tank to the test tank, through careful handling, to reduce stress. During the behavioral record, the tank was in an independent room, without any interference, to avoid the effects from surrounding area. After the fish were allowed 15 min of acclimation, the opaque partition was removed without excessive disturbance. Zebrafish behaviors were videotaped for 10 min by a digital camera (SONY HDR-SR11), which was mounted at the lateral site of the test tank, and an experienced observer analyzed the mirror-biting frequency.

### 4.11. Statistical Analysis

Data are expressed as the mean ± deviation (SD) for the parametric data and the mean ± standard error of the mean (SEM) for nonparametric data. Before statistical analysis was performed, ROUT method was used to identify the outlier. Then the Shapiro–Wilk normality test was applied to evaluate normality in all the datasets. The datasets showing normal distribution were analyzed by Student’s *t*-test. The dataset of the mirror-biting test with AC treatment was not normally distributed and was analyzed by Mann–Whitney test (Figure 7B). The raw data used for all statistical analyses can be found at https://tinyurl.com/oxytocinzebrafish, accessed on 1 June 2021. Statistical analysis was conducted by using GraphPad Prism 7.

## 5. Conclusions

In summary, our results demonstrated that the oxytocin system serves as a potential stress indicator that enables animals to adapt to environmental fluctuations. Oxytocin regulates physiological functions through the endocrine system and affects behavioral outcomes by innervating downstream nuclei in the brain. Under stress, behavioral change is a strategy through which animals adapt to extreme environments. Such behavioral changes may allocate energy metabolism, which improves animal survival with minimal adjustments to physiological and metabolic responses and ultimately increases the fitness of animals for environmental adaptation.

## Figures and Tables

**Table 1:** The information of primer sets used in the present study.

Genes	Primer Sequence (5′–3′)	Amplicon Size	EF	Ensemble ID
*oxyr*	F: TTCAGCATCCCGCAGGTTTA	123 bp	95.4%	ENSDART00000176856.2
	R: GCACTGGTCCCTCTTCGTCTT			
*oxyrl*	F: ACGCCCTTCTTCTTCGTTCAG	147 bp	98.4%	ENSDART00000064853.4
	R: TATTTCTCCAGTGCCTCTTACAGC			
*rpl13a*	F: CCTCGGTCGTCTTTCCGCTATTG	247 bp	95.2%	ENSDART00000180298.1
	R: CAGCCTGACCCCTCTTGGTTTTG			

F, forward primer; R, reverse primer; EF, efficiency of primer pair in semi-quantitative PCR and RT-qPCR.

## Data Availability

The raw data used for all statistical analyses can be found at https://tinyurl.com/oxytocinzebrafish, accessed on 1 June 2021.

## Abbreviations

AC	acidic
HA	high ammonia
DI	double-ionized
HPA	hypothalamic–pituitary–adrenal
GR	glucocorticoid receptor
MO	morpholino
AuNP-PVP	gold nanoparticles coated with PVP
PPa	the anterior part of the parvocellular preoptic nucleus
PPp	the posterior part of the parvocellular preoptic nucleus
TPp	the periventricular nucleus of the posterior tuberculum
Hc	the caudal zone of the periventricular hypothalamus
Vv	the ventral nuclei of the ventral telencephalic area
VM	the ventromedial thalamic nuclei
A	the anterior thalamic nuclei
PGm	the preglomerular nuclei
TPp	the periventricular nucleus of the posterior tuberculum
Cans	the commissura ansulata
IPN	the interpeduncular nucleus
TTBc	the crossed tecto-bulbar tract
PVN	the paraventricular nucleus of the hypothalamus
PFC	the prefrontal cortex
Dpf	day-post-fertilization
mPFC	the medial prefrontal cortex
CB1 receptor	cannabinoid receptor type-1
RE	the reuniens nuclei in the thalamus
RH	the rhomboid nuclei in the thalamus
CeL/C	the lateral and capsular division of the central amygdala
CeM	the central amygdala
dHbM	the medial subregion of the dorsal habenula
i/vIPN	the intermediate/ventral interpeduncular nucleus
dHbL	the lateral subregion of the dorsal habenula
d/iIPN	the dorsal/intermediate interpeduncular nucleus
DRN	the dorsal raphe nucleus
